# Off-label use of antipsychotic medications in psychiatric inpatients in China: a national real-world survey

**DOI:** 10.1186/s12888-021-03374-0

**Published:** 2021-07-27

**Authors:** Juan Wang, Feng Jiang, Yating Yang, Yulong Zhang, Zhiwei Liu, Xiaorong Qin, Xueqin Tao, Tingfang Liu, Yuanli Liu, Yi-lang Tang, Huanzhong Liu, Robert O. Cotes

**Affiliations:** 1 The Clinical Hospital of Chengdu Brain Science Institute, MOE Key Lab for Neuroinformation, University of Electronic Science and Technology of China, Chengdu, China; 2 The Fourth People’s Hospital of Chengdu, Chengdu Mental Health Center, 8 Hulixiyixiang, Jinniu District, Chengdu, China; 3 Institute of Health Yangtze River Delta, Shanghai Jiao Tong University, 1954 Huashan road, Xuhui district, Shanghai, China; 4 Department of Psychiatry, Chaohu Hospital of Anhui Medical University, 64 Chaohu North Road, Chaohu District, Hefei, China; 5 Department of Psychiatry, Anhui Psychiatric Center, 64 Chaohu North Road, Chaohu District, Hefei, China; 6 Department of Psychiatry, Fuyang Third People’s Hospital, Fuyang, Anhui China; 7 Institute for Hospital Management of Tsinghua University, K308 Tsinghuayuan District, Shenzhen, China; 8 School of Public Health, Chinese Academy of Medical Sciences and Peking Union Medical College, 5 Dongdansantiao, Dongcheng District, Beijing, 100730 China; 9 Department of Psychiatry and Behavioural Sciences, Emory University School of Medicine, 12 Executive Park Drive NE, Suite 150, Atlanta, GA 30329 USA; 10 Mental Health Service Line, Atlanta VA Medical Center, Decatur, GA 30033 USA

**Keywords:** Off-label, Antipsychotic, Psychiatric, China

## Abstract

**Background:**

The off-label use of antipsychotic medications is common in many countries, and the extent of such use in psychiatric inpatients in China has not been sufficiently studied. The purpose of this study was to survey the incidence and examine the correlates of off-label antipsychotic use in a large, nationally–representative sample in China.

**Methods:**

This study included discharged psychiatric patients between March 19 and 31, 2019 from 41 tertiary psychiatric hospitals across 29 provinces in China. Their socio-demographic and clinical data were collected and analyzed.

**Results:**

After excluding patients with schizophrenia spectrum disorder or bipolar disorder, 981 patients were included in the analysis. Overall, antipsychotics were prescribed to 63.2% (95%CI 60.2–66.2%) of the sample. Antipsychotics were used in a wide spectrum of psychiatric disorders, with the rate being the highest among patients with dissociative (conversion) disorders (89.9, 95%CI 83.0–94.8%), organic mental disorders (81.7, 95%CI 73.1–88.7%), dementia (79.0,95%CI 67.8–87.9%), obsessive-compulsive disorder (77.8, 95%CI 55.7–92.5%), mental disorders due to psychoactive substances (75.3,95%CI 64.7–84.2%), behavioural and emotional disorders with onset usually occurring in childhood and adolescence (71.4, 95%CI 45.5–90.1%), somatoform disorders (63.2, 95%CI 40.8%–82..2%), major depression disorder (53.7,95%CI 48.8–58.6%), anxiety disorder (38.8,95%CI 30.5–47.7%), and insomnia (25.0, 95%CI 8.5–28.9%). The top three most commonly used antipsychotics were olanzapine (29.1%), quetiapine (20.3%) and risperidone (6.8%), and their corresponding average doses were 9.04 ± 5.80 mg/day, 185.13 ± 174.72 mg/day, and 2.98 ± 1.71 mg/day, respectively. A binary logistic regression showed that younger age, having the Employee Health Insurance or Residents Health Insurance, having psychotic symptoms and requiring restraint during hospitalization were significantly associated with off-label use of antipsychotics.

**Conclusion:**

Off-label use of antipsychotics is very common in psychiatric inpatients in China, mainly with moderate-dose use of single agents. However, the efficacy and safety of this practice is uncertain for many diagnoses and for the elderly. Clinicians should be cautious about this practice while waiting for more research data.

## Background

The term “off-label prescribing” refers to the use of mediation for a diagnosis, age group, or biological condition (such as pregnancy) that is not an officially approved use of that medication, as determined by the relevant regulatory body in the country. Many factors contribute to off-label use of medication in psychiatry. Our field has not realized the promise of a personalized medicine approach [1]. We have an expanding, but incomplete biological understanding of the pathophysiology of nearly every mental illness [1], and current pharmacologic treatments generally act on a broad range of receptor systems in the brain. From a phenomenological standpoint, different individuals may have a wide range of different presentations despite having the same diagnosis, and the same person may have multiple, discrete diagnoses with overlapping symptoms [2–4]. Practically, there are a limited number of medications available for a relatively large number of mental disorders, and there is often a slow, expensive process for approval for a new indication which may disincentivize pharmaceutical companies [5].

Off-label prescribing in most settings is common and legal, and may be reasonable and necessary in several scenarios: 1) it gives prescribers an opportunity to provide their patients the latest possible treatment options (e.g. the evidence for a treatment may exist and may be compelling, but the mediation has not gone through the official approval process); 2) one of the medications within a particular drug class has been approved, but the medication of interest has not been approved; 3) current treatments options have failed and the patient may be facing a life-threatening situation or 4) due to social and institutional pressure, clinicians need to control the patient’s condition in a short period of time [2, 5, 6]. However, inappropriate, unjustified, or reflexive off-label prescribing may put patients at unnecessary risks for side effects, may not be efficacious, and may have medico-legal consequences.

Antipsychotic drugs have been widely used across the world and evidence suggests their use may be increasing in some countries [7]. Antipsychotics can cause a wide array of side effects, which necessitates their judicious use [8, 9]. Generally, antipsychotic medications are indicated for the treatment of schizophrenia and many have an indication for the treatment of at least one phase of bipolar disorder [10]. In recent years, specific antipsychotic medications have received other indications through the U.S.Food and Drug Administration. For example, risperidone and aripiprazole were approved to treat behavioural disturbance associated with autism spectrum disorders [11, 12] . Olanzapine combined with fluoxetine was approved for treatment-resistant depression [13] . Aripiprazole, quetiapine and quetiapine extended-release were approved as augmentation therapy for major depression disorder [12, 14, 15] . Additionally, aripiprazole was approved to treat Tourette’s syndrome [12.

However, many antipsychotic medications are used for conditions other than their officially approved indications. An incomplete list includes the following: behavioural and psychological symptoms of dementia, borderline personality disorders, anxiety disorder, depressive disorder, post-traumatic stress disorder, attention deficit-hyperactivity disorder, obsessive-compulsive disorder, eating disorders, substance use disorders, and insomnia [16, 17]. The rates of off-label antipsychotic prescribing vary throughout the world, but in some settings, the rates of off-label prescribing exceed the “on” label prescribing. For example, Graziul et al. analysed 781 million prescriptions of psychotropic drugs in the US, and they found the overall average off-label utilization rate of second-generation antipsychotics (SGAs) was 60.7% [18].

Data on off-label use of antipsychotics in China, the world’s largest country by population, are scarce. Based on data from two studies in Taiwan, the use of antipsychotics in patients with intellectual disability, increased from 7.2% in 1997 to 13.4% in 2007 [19], and the use in outpatients with anxiety disorders increased from 8.4% in 2005 to 9.1% in 2013 [20]. In a study from Hong Kong that included data from 256,903 patients, over half (52.5%) that received an antipsychotic received the antipsychotic for a nonpsychotic mental disorder [21].

Although these studies provided a glimpse into off-label antipsychotic utilization, there are limited in several aspects: they often only included a single study site, there were no detailed descriptions of antipsychotic prescriptions, and few studies examined factors associated with antipsychotics use (such as demographic and psychiatric symptoms). Furthermore, no such studies have been conducted in mainland China. Based on this gap in the literature, this study sought to quantify the utilization of off-label use of antipsychotics in patients from 41 tertiary psychiatric hospitals in mainland China. Our aim was to explore the frequency, demographic, and clinical correlates of off-label antipsychotic use in a large, nationally representative sample.

## Methods

### Participants

The National Health and Family Planning Commission (NHFPC) of China launched a national survey to understand health care trends and improve healthcare services in 2015. In the third (2017) and fourth (2019) iteration of the national surveys, psychiatric surveys were added. These results of this study were based on the 2019 survey data. In this survey, NHFPC selected 41 tertiary psychiatric hospitals to participate in the project. These hospitals are the best in China, in terms of resources and staffing composition in psychiatry. A well-designed and unified survey form was used, which included the demographic information and clinical data of the patients. The clinicians in each hospital filled in the survey form when the patient was discharged between March 19 and 30, 2019, regardless of sex, age, and diagnosis. Finally, all the forms were summarized and the data was entered. A total of 2665 patients completed the survey. Patients who were discharged against medical advice (AMA) (*N* = 264) and patients who were diagnosed with schizophrenia spectrum disorder or bipolar disorder (*N* = 1420) were excluded. Ultimately, a total of 981 patients were included in the data analysis.

According to the diagnosis of International Classification of Diseases -10th Edition (ICD-10), among the 981 patients, there were 391 cases of major depressive disorder (39.1%) (F32–33), 121 cases of anxiety disorder (12.3%) (F41), 99 cases of dissociative (conversion) disorder (10.1%) (F44), 93 cases of organic mental disorders (9.5%) (F04–09), 73 cases of mental disorder due to psychoactive substances (7.4%) (F10–19), 62 cases of dementia (6.3%) (F00–03), 19 cases of somatoform disorder (1.9%) (F45), 18 cases of obsessive-compulsive disorder (1.8%) (F42), 16 cases of insomnia (1.6%) (F51), 14 cases of behavioural and emotional disorders with onset usually occurring in childhood and adolescence (1.4%) (F90–98), and 75 cases of others (a miscellaneous group) (10.4%). The miscellaneous group included a small number of cases of persistent mood disorder (F34), reaction to severe stress and adjustment disorders (F43), eating disorder (F50), disorders of adult personality and behaviour (F60–69), mental retardation (F70–79), and unspecified mental disorder (F99).

### Data collection

For all patients, socio-demographic data and clinical data were retrieved from the electronic medical record. Clinical data included diagnosis, clinical symptoms, and treatments, such as duration of illness, number of hospitalizations, whether they were involuntarily hospitalized, whether they received restraints while hospitalized, and whether they received electroconvulsive therapy (ECT) treatment. Medical insurance was divided into three categories: Employee Health Insurance, Resident Health Insurance, and others (which included self-pay, commercial insurance and Medical Assistance). The reimbursement rate of the Employees Health Insurance is higher than that of the Resident Health Insurance. The Global Assessment of Function (GAF) score was recorded at admission to assess the psychosocial function of the patients [22]. Whether the patient used antipsychotics at discharge, as well as the type and dose of antipsychotics was recorded. We used the DDD (defined daily doses) method to convert the antipsychotic dose to chlorpromazine equivalents [23].

### Statistical analysis

Patients were divided into two groups based on whether or not they were prescribed an antipsychotic medication at discharge. First, the socio-demographic and clinical variables of the two groups were compared. Pearson’s Chi-square test was used to compare categorical variables and Mann-Whitney U test was used to compare continuous variables that were not normally distributed. Second, the utilization rate of antipsychotics for each diagnosis was calculated. Third, the proportion and dosage of each antipsychotic in the total sample and for different disorders was calculated. Fourth, a binary logistic regression was performed to examine the correlates of off-label antipsychotic use, using antipsychotic prescription status as a dependent variable (reference = not using antipsychotics) and all remaining socio-demographic and clinical variables as independent variables. All statistical analyses were performed using the SPSS 23.0. We used two-sided tests where applicable, and *P* < 0.05 was defined as statistically significant.

## Results

### Comparison of socio-demographic and clinical data of psychiatric patients with and without antipsychotics

A total of 981 psychiatric patients were included in the analysis. There were 392 (40.0%) males and 589 (60.0%) females, with an average age of 46.72 ± 19.97 years. At the time of survey completion, 63.2% (620/981) patients were prescribed either one or more antipsychotic medications. Of the group that received antipsychotic medications, 89.0% (552/620) patients received a single antipsychotic drug, and 11.0% (68/620) patients received two or more antipsychotic drugs. Among these patients, 15.6% (153/981) of them used a combination of mood stabilizers and antipsychotics, 33.3% (327/981) used a combination of antidepressants and antipsychotics, and 24.9% (244/981) used a combination of sedatives/hypnotics (including benzodiazepines) and antipsychotics. The socio-demographic and clinical characteristics of patients with and without antipsychotics were shown in Table 1.

**Table 1:** Comparison of socio-demographic and clinical data of psychiatric patients with and without APs

	Total (*N* = 981)	APs (*N* = 620)	No APs (*N* = 361)	*Z/χ2*	*p*
Male	392 (40.0%)	262 (42.3%)	130 (36.0%)	3.71	0.054
Age (years ± SD)	46.72 ± 19.97	46.02 ± 20.55	47.91 ± 18.89	−1.84	0.066
Marital status					
Married	625 (63.7%)	371 (59.8%)	254 (70.4%)	11.12	0.004
Single	240 (24.5%)	166 (26.8%)	74 (20.5%)		
Divorced/widowed	116 (11.8%)	83 (13.4%)	33 (9.1%)		
Education background					
Uneducated/primary/ middle school	499 (50.9%)	335 (54.0%)	164 (45.4%)	6.76	0.034
Senior high/ vocational school	270 (27.5%)	160 (25.8%)	110 (30.5%)		
College school or above	212 (21.6%)	125 (20.2%)	87 (24.1%)		
Medical insurance					
Employee medical insurance	391 (39.9%)	243 (39.2%)	148 (41.0%)	11.57	0.003
Residents medical insurance	423 (43.1%)	288 (46.5%)	135 (37.4%)		
Others	167 (17.0%)	89 (14.4%)	78 (21.6%)		
Duration of illness (years)	7.14 ± 9.13	7.22 ± 9.23	7.00 ± 8.98	−0.15	0.883
GAF scores at admission	52.57 ± 18.59	49.71 ± 18.55	57.48 ± 17.63	−6.96	< 0.001
First hospitalization (yes, %)	570 (58.1%)	341 (55.0%)	229 (63.4%)	6.67	0.010
Psychotic symptoms (yes,%)	337 (34.4%)	294 (47.4%)	43 (11.9%)	127.56	< 0.001
Involuntary admission	206 (21.0%)	169 (27.3%)	37 (10.2%)	39.79	< 0.001
Restrained during hospital (yes, %)	145 (14.8%)	128 (20.6%)	17 (4.7%)	46.00	< 0.001
Agitation (yes, %)	162 (16.5%)	138 (22.3%)	24 (6.6%)	40.32	< 0.001
Suicidality and self-injurious behavior (yes, %)	41 (4.2%)	31 (5.0%)	10 (2.8%)	2.83	0.092
Received ECT treatment (yes, %)	96 (9.8%)	67(10.8%)	29 (8.0%)	1.99	0.159

*APs* antipsychotics, *GAF* global assessment function scale, *ECT* electroconvulsive therapy

### The utilization rate of antipsychotics in different disorders

**Fig. 1:**
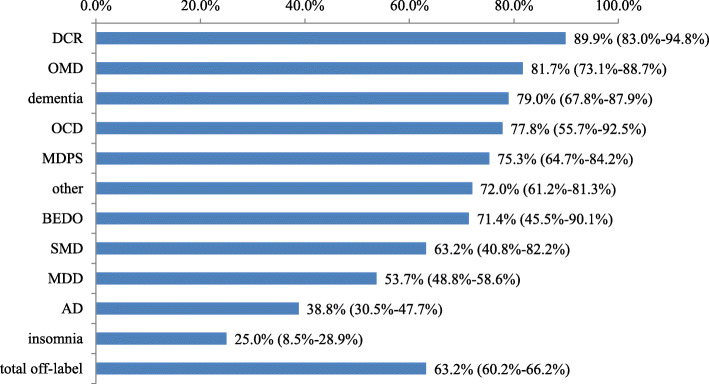
The use rates of APs in different diseases at discharge (N=981). Data show the proportion of antipsychotics used for a certain diagnosis, with 95% confidence intervals in parentheses. APs: antipsychotics; DCR: dissociative (conversion) disorders; OMD: organic mental disorders; OCD: obsessive-compulsive disorder; MDPS: mental disorders due to use of psychoactive substances; BEDO: behavioural and emotional disorders with onset usually occurring in childhood and adolescence; SMD: somatoform disorders; MDD: major depression disorders; AD: anxiety disorder.

The utilization rate of antipsychotics for each diagnostic category can be seen in Fig. 1. The highest utilization rate of antipsychotics was dissociative (conversion) disorder (89.9, 95%CI 83.0–94.8%, 89/99), followed by organic mental disorders (81.7, 95%CI 73.1–88.7%, 76/93), dementia (79.0, 95%CI 67.8–87.9%, 49/62), obsessive-compulsive disorder (77.8, 95%CI 55.7–92.5%,14/18) and the lowest was insomnia (25.0,95%CI 8.5–28.9%, 4/16). The rate of antipsychotic medications was also relatively high in patients with major depression disorder (53.7, 95%CI 48.8–58.6%, 210/391) and anxiety disorder (38.8, 95%CI 30.5–47.7%, 47/121).

### Types and utilization rates of different antipsychotics in psychiatric patients at discharge

As shown in Table 2, the most commonly used antipsychotics overall were olanzapine (29.1%), quetiapine (20.3%) and risperidone (6.8%). SGAs made up the preponderance of antipsychotics prescribed (97.6%) and first-generation antipsychotics (FGAs) were used only in 2.4%. For patients with major depressive disorders, the top three most frequently prescribed antipsychotics were olanzapine (23.8%), quetiapine (19.9%) and aripiprazole (5.9%). Patients with anxiety disorders were most commonly treated with quetiapine (17.4%) and olanzapine (15.7%). Olanzapine was often used in patients with dissociative (conversion) disorders (55.6%), organic mental disorders (44.1%), and somatoform disorders (42.1%). Over half of patients with obsessive-compulsive disorder were on aripiprazole (55.6%). Patients with behavioural and emotional disorders with onset usually occurring in childhood and adolescence were most commonly treated with aripiprazole (42.9%).

**Table 2:** Antipsychotic preferences for different disorders at discharge (n = 981)

	Total	MDD	AD	DCR	OMD	MDPS	Dementia	SMD	OCD	Insomnia	BEDO	Other
	N = 981	*N* = 391	*N* = 121	*N* = 99	*N* = 93	*N* = 73	N = 62	*N* = 19	*N* = 18	*N* = 16	N = 14	*N* = 75
Olanzapine	29.1%	23.8%^a^	15.7%	55.6%	44.1%	30.1%	27.4%	42.1%	5.6%	12.5%	14.3%	33.3%
Quetiapine	20.3%	19.9%^b^	17.4%	21.2%	18.3%	23.3%	38.7%	15.8%	11.1%	12.5%	21.4%	14.7%
Risperidone	6.8%	2.0%	–	11.1%	15.1%	13.7%	14.5%	5.3%	5.6%	–	14.3%	14.7%
Aripiprazole	6.6%	5.9%^b^	2.5%	5.1%	5.4%	4.1%	1.6%	–	55.6%	–	42.9%	12.0%
Clozapine	1.9%	2.0%	2.5%	2.0%	1.1%	1.4%	1.6%	–	–	–	7.1%	2.7%
Other SGAs ^c^	3.2%	2.3%	0.8%	7.1%	5.4%	5.5%	–	5.3%	–	–	–	5.3%
FGAs ^d^	2.4%	1.8%	0.8%	3.0%	5.4%	4.1%	3.2%	5.3%	–	–	–	2.7%

^a^Olanzapine and fluoxetine in combination was approved by FDA for treatment of treatment resistant depression, but not approved by CFDA

^b^Aripiprazole, quetiapine and quetiapine XR was approved by FDA as augmentation therapy for treatment resistant depression, but not approved by CFDA

^c^Other SGAs: including amisulpride, ziprasidone, paliperidone and perospirone

^d^FGAs: including perphenazine, haloperidol, sulpiride and chlorpromazine

*APs* antipsychotics, *MDD*: major depression disorders, *AD*: anxiety disorder, *DCR*: dissociative (conversion) disorders, *OMD* organic mental disorders, *MDPS* mental disorders due to psycho active substances, *SMD* somatoform disorders, *OCD* obsessive-compulsive disorder, *BEDO* behavioural and emotional disorders with onset usually occurring in childhood and adolescence

### Doses of different antipsychotics in psychiatric patients at discharge

As shown in Table 3, the mean antipsychotic dose for the entire sample, as measured by chlorpromazine equivalent, was 231.56 ± 191.49 mg/day. The chlorpromazine equivalent for dissociative (conversion disorders) (336.22 ± 200.7 mg/day) was the highest, followed by miscellaneous group (308.65 ± 247 mg/day), then organic mental disorders (285.78 ± 167.56 mg/day). The mean chlorpromazine equivalents used for insomnia (150 ± 129.9 mg/day), anxiety disorder (144.32 ± 144.67 mg/day) and dementia (138.02 ± 131.22 mg/day) were the lowest. The antipsychotic doses varied widely for different disorders. For example, the average dose of olanzapine was 9.04 ± 5.80 mg/day, with a range from 5 mg/day (organic mental disorders) to 12.5 ± 3.54 mg/day (behavioural and emotional disorders with onset usually occurring in childhood and adolescence). The average dose of quetiapine was 185.13 ± 174.72 mg/day, with a range from 100 mg/day (insomnia) to 333.33 ± 251.66 mg/day (behavioural and emotional disorders with onset usually occurring in childhood and adolescence).

**Table 3:** Doses of different APs in different disorders at discharge (*N* = 603^a^)

	Chlorpromazine equivalent	Olanzapine	Quetiapine	Risperidone	Aripiprazole	Clozapine
Total Sample	231.56 ± 191.49	9.04 ± 5.80	185.13 ± 174.72	2.98 ± 1.71	9.13 ± 6.32	86.05 ± 117.15
MDD	199.06 ± 192.67	7.66 ± 5.62	180.08 ± 175.44	3.14 ± 1.57	10.33 ± 7.16	95.94 ± 164.72
AD	144.32 ± 144.67	6.97 ± 5.75	111.31 ± 121.38	–	5.83 ± 3.82	85 ± 99.62
DCR	336.22 ± 200.7	11.18 ± 5.79	266.43 ± 210.70	3.32 ± 1.49	16 ± 9.62	75 ± 35.36
OMD	285.78 ± 167.56	10.63 ± 5.49	232.35 ± 194.41	2.73 ± 1.42	6..5 ± 3.35	225
MDPS	214.82 ± 140.55	9.75 ± 5.19	208.09 ± 143.54	2.7 ± 1.06	7.5 ± 3.54	25
Dementia	138.02 ± 131.22	6.98 ± 5.53	103.33 ± 95	1.47 ± 1.76	10	12.5
SMD	206.56 ± 132.97	6.56 ± 3.99	200 ± 180.28	1	–	–
OCD	168.21 ± 75.64	5	250 ± 70.71	3	8.25 ± 4.42	–
Insomnia	150 ± 129.9	10	100	–	–	–
BEDO	284.50 ± 185.73	12.5 ± 3.54	333.33 ± 251.66	3.5 ± 0.7	7.5 ± 2.74	25
Other	308.65 ± 247	9.89 ± 6.52	247.22 ± 251.39	4.55 ± 1.95	6.88 ± 6.23	87.5 ± 53.03

The data in the table was described with Mean ± Standard deviation

^a^There are 17 patients whose data of APs dose were not available

*APs* antipsychotics, *MDD* major depression disorders, *AD* anxiety disorder, *DCR* dissociative (conversion) disorders, *OMD* organic mental disorders, *MDPS* mental disorders due to psychoactive substances, *SMD* somatoform disorders, *OCD* obsessive-compulsive disorder, *BEDO* behavioural and emotional disorders with onset usually occurring in childhood and adolescence

### Logistic regression of demographic and clinical factors of patients with antipsychotics

Table 4 shows the results of the logistic regression. Younger age, having the Employee Health Insurance or Residents Health Insurance, presence of psychotic symptoms and requiring an episode of restraint during hospitalization were predictors of off-label use of antipsychotics.

**Table 4:** Logistic analysis of demographic and clinical factors of using APs in psychiatric patients (*N* = 981)

	P	OR	95% CI
Male (ref.female)	0.903	0.98	0.72–1.33
Age	**0.030**	0.99	0.97–0.99
Marital status (ref.married)			
Single	0.924	0.98	0.60–1.59
Divorced/widowed	0.146	1.44	0.88–2.34
Education background (ref. uneducated /primary / middle school)			
Senior high/ vocational school	0.235	0.81	0.57–1.15
College school or above	0.094	0.71	0.48–1.06
Medical insurance (ref. others)			
Employee Medical Insurance	**0.022**	1.65	1.07–2.54
Residents Medical Insurance	**0.016**	1.66	1.10–2.50
Duration of illness (years)	0.884	1.00	0.98–1.02
GAF scores at admission	0.059	0.99	0.98–1.00
First hospitalization (yes, %)	0.126	0.79	0.58–1.07
Psychotic symptoms (yes,%)	**< 0.001**	4.90	3.34–7.19
Involuntary admission	0.218	1.34	0.84–2.13
Restrained during hospital (yes, %)	**0.014**	2.34	1.19–4.63
Agitation (yes, %)	0.556	1.20	0.65–2.20
Suicidality and self-injurious behavior (yes, %)	0.836	1.09	0.47–2.52
Received ECT treatment (yes, %)	0.262	1.34	0.81–2.24

*APs* antipsychotics, *GAF* global assessment function, *ECT* electroconvulsive therapy

## Discussion

In a large sample of psychiatric patients discharged from inpatient units in mainland China (excluding individuals with schizophrenia spectrum disorder or bipolar disorder), we found that off-label use of antipsychotic medication was common and occurred for almost all psychiatric disorders. In the total sample, 63.2% of psychiatric patients were prescribed at least one off-label antipsychotic drugs at the time of discharge. The most commonly used antipsychotic drugs were olanzapine (29.1%) and quetiapine (20.3%), with an average daily dose of 9.04 ± 5.80 mg/day and 185.13 ± 174.72 mg/day, respectively.

Our overall rate of off-label use of antipsychotics (63.2%) was consistent with the finding in the US (60.7%) by Graziul.et al. [18]. Our rates of off-label use in specific diagnostic categories add a unique contribution and differ, in some cases, from the existing literature. For example, in patients with dementia, the rate in our study (79.0%) was higher than those reported by Harding.et al. (28.1%) [24] and Davids.et al. (67.3%) [25]. Similar trends are found with other diagnoses: for organic mental disorders, 81.7% (our report, hereafter) vs approximately 50% [25]; anxiety disorders, 38.8% vs 6.9–14.5% [26]; depressive disorders, 53.7% vs 39.4% [27]; insomnia, 25% vs 12.1% [28]. These differences could be explained by variation in patient populations, prescribing practices, economic considerations, and institutional policies. We found a high rate of the use of antipsychotics in dissociative (conversion) disorders (89.9%), organic mental disorders (81.7%), and dementia (79.0%). In our sample, these three groups usually presented to the inpatients unit with psychotic symptoms, suggesting psychiatrists may have routinely added antipsychotic medication as treatment.

Why are antipsychotic medications used so broadly for psychiatric disorders? Based on the pharmacodynamic properties of antipsychotics, we propose three possible explanations. First, SGAs often affect a broad range of activities at multiple neurotransmitter systems [29]. Some SGAs (such as quetiapine and clozapine) possess a fairly weak affinity to the dopamine D2 receptors, so their antipsychotic efficacy may not be evident until used at a higher dose. SGAs also antagonize 5-HT 2a and 2c receptors generally at lower doses, which may result in their therapeutic properties for anxiety and depression [29]. Second, many mental disorders have multi-dimensional symptoms (such as positive, negative, cognitive symptoms of schizophrenia) as well as comorbidity, such as patients with organic mental disorders with psychotic symptoms, and demented patients with behavioural disorders [30, 31]. Third, according to the drug-center model of drug action, the sedation, emotional indifference and akinesia induced by antipsychotics could be beneficial for acute psychosis, and these effects may also reduce agitation, anxiety, and insomnia which occur across many diagnoses [32].

In our sample, the average chlorpromazine equivalent dose was moderate on average (231.56 ± 191.49 mg/day), but lower compared to the dose used for schizophrenia. This is consistent with the rationale above that SGAs likely affect different receptor systems based on the dose used, and may have therapeutic properties for anxiety and depression at lower doses. In contrast, based on data from multiple studies, chlorpromazine equivalents for people with schizophrenia were mostly greater than 400 mg/day [33–35]. When looking at specific antipsychotics in our sample, this principle held up. For example, the average dose of olanzapine for the treatment of major depression disorder was 7.66 ± 5.62 mg/day (compared to 10–20 mg for schizophrenia [36]), and for quetiapine in the treatment of anxiety disorder, the dose was 111.31 ± 121.38 mg/day (compared to 300–750 mg for schizophrenia [36]). However, in dissociative (conversion) disorders, organic mental disorders, other disorders accompanied by psychotic symptoms, behavioural disorders, or aggression, the antipsychotic doses approached ones more commonly used for schizophrenia.

While antipsychotics may have a role in treating anxiety, depression, insomnia, and other conditions, clinicians must consistently evaluate and update the risk-benefit ratio for each decision in an evidence-base that is constantly being updated. In a comprehensive review summarizing the literature on off-label antipsychotic use, the Agency for Healthcare Research and Quality concluded: 1) for elderly patients with dementia with behavioural and psychological disturbances, aripiprazole, olanzapine and risperidone had small but statistically significant effects, although the risk of death, stroke, extrapyramidal symptoms, and urinary tract symptoms was significantly increased, 2) quetiapine had a small benefit for anxiety disorder, 3) risperidone could improve the response for obsessive-compulsive disorder, 4) SGAs were not effective for eating disorders, personality disorders, and substance use disorders, and 5) the data on the use of antipsychotics to treat insomnia was inconclusive [37, 38]. Overall, there is insufficient evidence on the efficacy and safety for most off-label applications of antipsychotics.

We found that the following factors were significantly associated with off-label antipsychotic use: younger age, having the Employee Health Insurance or Residents Health Insurance, having psychotic symptoms, and requiring restraints during hospitalization. The findings are similar to those of other studies [39–41]. Young patients may be more prone to agitated or aggressive behaviour, and are more likely to be on antipsychotic drugs, despite their diagnosis [42, 43]. In China, most antipsychotic drugs are covered by health insurances. When insured, patients would be more able to afford the costs of these drugs after hospital discharge. Restraints during hospitalization indicate that the patient may have exhibited aggressive, impulsive, or agitated behaviour. Patients with psychotic symptoms, impulsive behaviour and agitation were more likely to be treated with antipsychotic drugs at a relatively high dose.

There are several limitations to this study. First, the study only included patients from tertiary psychiatric hospitals which receive referrals from other hospitals and often (not always) had more acute and complicated patients. Therefore the findings may not be generalizable to other patients, especially those in rural settings or in forensic settings. Second, this was a cross-sectional survey, so it was not possible to infer causal relationships. Third, due to cross-sectional nature of the survey, the dose of antipsychotic was only taken at discharge. It was not clear if an off-label antipsychotic medication was stopped prior to discharge (and therefore not captured), whether the dose will be changed after discharge, or if the antipsychotic drug will be stopped after discharge.

## Conclusion

This study reported the high frequency of off-label antipsychotic medications among in patients at the time of discharge from psychiatric hospitals. The most commonly used antipsychotic drugs were olanzapine and quetiapine, and their dosage was relatively low. Although off-label use is common and sometimes necessary, the efficacy and safety of this strategy is uncertain for some diagnoses and for the elderly. Clinicians should be cautious about this practice and more research is needed.

## Data Availability

The datasets used and/or analysed during the current study are available from the corresponding author on reasonable request.

## Abbreviations

SGAs: Second-generation antipsychotics
NHFPC: National Health and Family Planning Commission
AMA: Against medical advice
ICD-10: International Classification of Diseases -10th Edition
ECT: Received electroconvulsive therapy
GAF: Global Assessment of Function scale
FGAs: First-generation antipsychotics
